# Editorial: E3 Ubiquitin Ligases: From Structure to Physiology

**DOI:** 10.3389/fphys.2020.621053

**Published:** 2020-11-26

**Authors:** Julien D. F. Licchesi, Heike Laman, Fumiyo Ikeda, Victor M. Bolanos-Garcia

**Affiliations:** ^1^Department of Biology and Biochemistry, University of Bath, Bath, United Kingdom; ^2^Department of Pathology, University of Cambridge, Cambridge, United Kingdom; ^3^Division of Inflammation and Proteostasis, Medical Institute of Bioregulation, Kyushu University, Fukuoka, Japan; ^4^Department of Biological and Medical Sciences, Oxford Brookes University, Oxford, United Kingdom

**Keywords:** E3 ubiquitin ligases, really interesting new gene (RING), HECTs (Homologous to the E6AP carboxyl terminus), RING-in between-RING (RBR), SCF (Skp1, Cullin, F-box protein), ubiquitin chain assembly, cancer, neurological disorders

Protein ubiquitination has emerged as a central regulatory mechanism of eukaryotic cells that affects multiple cellular processes and is critical for timely protein degradation and signal transduction. The topological nature of the assembled ubiquitin chain largely dictates the function of the ubiquitinated protein and the cellular outcome. The molecules responsible for the post-translational modification of protein substrates with ubiquitin comprise E1 ubiquitin-activating, E2 ubiquitin-conjugating, and E3 ubiquitin ligating enzymes. While deubiquitinating (DUB) enzymes which remove ubiquitin chains have received a lot of attention, especially in the field of drug discovery where some DUB inhibitors are approaching clinical trials, E3 ubiquitin ligases are only recently coming into the limelight.

Despite impressive advances in the structural to physiological understanding of E3 ubiquitin ligases, important aspects of E3s structure to function remain obscure. Undoubtedly, an enhanced knowledge of the conformational dynamics, macromolecular interactions, and functional integration of E3 ligases into cellular pathways will illuminate the precise roles of E3 ligases in aberrant signaling processes. This will pave the way toward rationally manipulating these enzymes for therapeutic intervention in many diseases that represent important health and societal challenges. Targeted protein degradation has emerged as a powerful approach for the removal of proteins driving human diseases. Amongst these, PROTACs, LYTACs, and AUTACs are technologies that harness the endogenous protein ubiquitination machinery and target proteins for degradation by the proteasome, lysosome, or through autophagy (Sakamoto et al., 2001; Takahashi et al., 2019; Banik et al., 2020). Given these recent advances, a more detailed understanding of E3 ubiquitin ligase functions and their modes of action are paramount in order to fully exploit these endogenous cellular waste recycling plants to improve human health.

The contributors to this Topic discuss how the integration of cellular, chemical, biophysical, and structural biology methods with “omics” approaches have clarified important aspects of E3 ubiquitin ligases activity, function, and mode of regulation. Such insights include the mode of cooperation of E3s with other enzymes for ubiquitin chain initiation and elongation, the precise positioning of the donor and acceptor ubiquitin sites, and the principles underpinning substrate-assisted catalysis. The subtle mechanistic and ligand-recognition variations that are inscribed in a spatial and temporal framework, confer these enzymes with great substrate selectivity and specificity. Excitingly, all the families of E3 ubiquitin ligases, including Really interesting Genes (RING), HECTs (Homologous to the E6AP carboxyl terminus), and RING-in between-RING, are represented in this Research Topic. We would also like to bring to the attention of readers, the Frontiers in Chemistry Research Topic on “Probing the Ubiquitin Landscape” which focus on new chemical biology tools and techniques to further dissect the role of protein ubiquitination (Mulder et al., 2020).

Progressing from E3 ligases structure to physiology, Deol et al. summarize our current understanding of the key molecular principles of ubiquitin chain assembly and discuss current evidence that supports two alternative, mechanistic models of polyubiquitylation: a “sequential addition” model wherein one ubiquitin unit is transferred at a time to a growing substrate-linked ubiquitin and an “en bloc” model in which ubiquitin units are assembled prior to their transfer onto a substrate. They also discuss how the weak and dynamic nature of the interactions represents a challenge to establish the trajectory of the functional enzyme-substrate complexes.

Weber et al. review HECT-containing E3 ligases and explain the reasons manipulating their catalytic activity is challenging include their transient interactions and low binding affinities with their substrates, as well as the redundancy among E3 ligases, where depending on the cellular context, a specific substrate may be modulated by several E3s. They argue that although the high conservation of the HECT domain within the HECT family means it may be difficult to develop specific inhibitory compounds, a greater understanding of the structural features and the underlying ubiquitination mechanisms used by different HECT E3 ligases remains an important biological question with the potential to unveil new opportunities for therapeutic intervention. Mayor and collaborators investigate the HECT ubiquitin ligase, UBE3A. Mutations in *Ube3a* cause Angelman and Prader-Willi syndromes (Wheeler et al., 2017; Harris and Stafford, 2020), both of which have no cure. Elu et al. identified the UBE3A-dependent ubiquitination sites and ubiquitin chain types formed on DDI1, a proteasome receptor. This contribution is an important step toward the full characterization of the UBE3A-dependent ubiquitination pathway that provides new insight into the molecular basis of rare neurological disorders. Some of the most poorly understood E3 ligases are the HERCs (homologous to the E6AP carboxyl terminus and regulator of chromosome condensation 1 (RCC1)-like domain-containing proteins), which are divided into large and small subfamilies. García-Cano et al. describe the high structural complexity of large-HERC family members and their important functions in diverse physiological processes, including cell proliferation, neuronal development, DNA repair, and inflammation. They also discuss the ways in which dysregulation of large-HERC ubiquitin ligases can lead to neurological disorders and cancer.

Mdm2 and its homolog MdmX (also known as Mdm4) are two RING E3 ubiquitin ligases that exert oncogenic activity. A precise understanding of the nature of Mdm2-MdmX interactions can be critical to exploiting them as potential therapeutic targets for reactivation of p53 function in tumors. Kosztyu et al. report a systematic mutational analysis of human Mdm2 that included the exchange of segments of its RING domain with the corresponding MdmX regions in order to identify the molecular features that determine their differential ability to forms dimers. Interestingly, the Mdm2 single substitution C449N blocked the ability of this protein to form a heterodimer with MdmX, but it did not disrupt Mdm2 RING self-association to form homodimers. Taken together, the studies suggest that the effect of certain conserved amino acid residues on Mdm2 homodimers and Mdm2-MdmX heterodimers formation is context-specific and possibly not entirely structurally equivalent. In contrast to HECT ligases, RING E3s do not have intrinsic catalytic activity *per se* and instead rely on adaptor molecules for the recruitment of substrate proteins (Deshaies and Joazeiro, 2009). Rathje et al. report an unanticipated function of Fbxo7, the substrate-recognition subunit of an SCF-type ubiquitin E3 ligase complex, in male germ cell cytoplasmic remodeling. They show that Fbxo7-deficient male mice were completely sterile despite successful meiosis, nuclear elongation and exclusion of histones from chromatin. At the same time, Fbxo7 mutant mice exhibited a sterility phenotype that has not been described before, where total death and phagocytosis of all condensing spermatids occurred in the absence of typical hallmarks of spermatid apoptosis. A previous, independent report on the fruit fly Fbxo7 ortholog nutcracker (ntc) shown to cause sterility at a similar stage of germ cell development, indicates a conserved requirement for Fbxo7 across species. The Fbxo7 mutant mice represent a valuable new animal model for the study of late spermiogenic, cell and tissue remodeling, and phagocytic events in germ cell development. Yoshida et al. focus their review on lectin-type F-box proteins, which recognize cytosolic sugar chains as markers of unwanted proteins and organelles that mediate aberrant or harmful signals and trigger ubiquitination, thus ensuring homeostasis. They concluded that elucidation of the molecular mechanisms underlying induction of sugar-recognizing F-box proteins and promotion of SCF complex formation by various stimuli is crucial for a deeper understanding of F-box proteins functions and of cytosolic sugar chains. The RING family of E3 ligases includes over 600 enzymes clustered into TRIM, UBR and cullin-RING ligases. Zanchetta and Meroni discuss MID1 and MID2, two members of the TRIpartite Motif (TRIM) family of RING E3 ligases, the overexpression of which is associated with lung adenocarcinoma, prostate and breast cancer. They review the emerging evidence supporting the involvement of MID1 and MID2 in the regulation of cytokinesis through MID1/MAD2 dynamic association with the proteins Astrin, BRAF35, and PP2A and how defects in the assembly of these complexes impairs cytokinesis, leading to severe adverse outcomes, including embryo development defects and cancer.

Approximately 50% of cancer patients receive treatment with ionizing radiation (radiotherapy) at some point during their cancer treatment. Fouad et al. present an analysis of how radiotherapy efficacy could be improved in combination with small size drugs that regulate the ubiquitin-proteasome system (UPS), some of which are currently undergoing clinical trials. The cullin family of E3 ligases is involved in several pathways of immune responses which can be exploited to modulate localized radiotherapy to induce out-of-target anti-tumor effects leading to tumor regression at non-irradiated metastatic sites, a phenomenon known as the abscopal effect. Fouad et al. go one step further and discuss the therapeutic approaches to target CRLs that have potential use in the clinic, including proteolysis targeting chimeras (PROTACs) of CRL adaptors that are only activated upon ionizing radiation or under a specific stimulus that mimic the tumor's microenvironment such as hypoxia that ultimately aim to improve patient survival.

Lescouzères and Bomont review the role of the Cullin 3-RING E3 ligase Gigaxonin in neurological diseases. Gigaxonin mutation are most commonly associated with Giant Axonal Neuropathies (GAN), a rare neurodegenerative disease which in the most severe cases has a fatal outcome usually before the third decade. Phenotypically, GAN results in enlarged axons which are filled with abnormal neurofilaments. The authors provide a comprehensive review of the roles and functions of Gigaxonin, including its known substrates, in cytoskeleton architecture, signal transduction through Hedgehog signaling and autophagosome production. The diversity in terms of substrates and cellular processes regulated by Gigaxonin further emphasizes the challenges associated with assigning a specific mechanism as disease-causing which currently limits the development of new therapies.

The RING-in between-RING (RBR) type of E3 ubiquitin ligases ubiquitinate substrates via a RING-HECT hybrid manner, and there are 14 RBR ligases known in humans. Lawrence et al. discuss about one of the RBR ligases, Natural Killer Lytic-Associated Molecule (NKLAM)/RNF19B in the regulation of innate immunity. NKLAM was originally identified by the authors as an IFNβ-induced gene in a human natural killer cell line. NKLAM regulates immune signaling cascades of STAT and NF-κB in immune cells, thus regulating cytokine production. Through its important roles of NKLAM in immunity, NKLAM shows anti-tumor function *in vivo*. More recent studies revealed that NKLAM regulates not only immunity, but also autophagy and responses to ER stress. The role of the ubiquitin ligase activity of NKLAM in innate immunity or other biological functions is yet to be discovered.

Finally, Escobar-Henriques and Joaquim discuss the role of E3 ubiquitin ligases in the regulation of mitochondrial quality control pathways. Mitochondria are highly dynamic organelles that constantly undergo anchoring, fission, transport, and membrane fusion. The latter process involves membrane remodeling in which the highly conserved proteins mitofusins (MFN1 and MFN2 in mammals and Fzo1 in yeast) play a central role. The authors review the ubiquitin E3 ligases that modify mitofusins in response to a variety of cellular inputs. They focus their analysis on the mitochondrial RING E3 ubiquitin ligases March5 and Mul1; Gp78, a ligase associated to the ER; and the cytosolic enzymes the RING MGRN1, the HECT ligase HUWE1 and the RBR E3 Parkin, all of which ubiquitylate mitofusins. The elucidation of molecular details underpinning mitofusins' modifications by E3 ligases should pave the way to develop new therapeutic approaches for the treatment of neurodegenerative, cardiovascular and obesity-associated disorders. The authors also provide novel insights on the roles of ubiquitin-dependent mechanisms in the regulation of inter-organelle membrane contact sites, a field which is revolutionizing our view of organelle biology (Scorrano et al., 2019).

In summary, this Research Topic highlights important discoveries in the structure to physiology understanding of E3 ubiquitin ligases, setting the stage to address important yet unanswered questions in the ubiquitin signaling field. These include the determination of the 3D structure of full length E3 ubiquitin ligases, which is now achievable, as the cryo-EM structure of the HECT ligase HUWE1 has shown (Hunkeler et al., 2020); the definition of the entire human ubiquitome; the mechanisms underlying substrate recognition and processing; the regulation of protein ubiquitination in time and space and tissue; the molecular basis for how deregulation of E3 ubiquitin ligases lead to disease. Addressing these questions will be instrumental in order to develop innovative therapeutics.

## Author Contributions

All authors listed have made a substantial, direct and intellectual contribution to the work and approved it for publication.

## Conflict of Interest

The authors declare that the research was conducted in the absence of any commercial or financial relationships that could be construed as a potential conflict of interest.
